# From Normal to Obesity and Back: The Associations between Mitochondrial DNA Copy Number, Gender, and Body Mass Index

**DOI:** 10.3390/cells8050430

**Published:** 2019-05-09

**Authors:** Daria Skuratovskaia, Larisa Litvinova, Maria Vulf, Pavel Zatolokin, Konstantin Popadin, Ilia Mazunin

**Affiliations:** 1 Laboratory of Immunology and Cell Biotechnology, Immanuel Kant Baltic Federal University, Kaliningrad 236016, Russia; larisalitvinova@yandex.ru (L.L.); mary-jean@yandex.ru (M.V.); endozapa@gmail.com (P.Z.); 2 Department of Reconstructive and Endoscopic Surgery, Kaliningrad Regional Hospital, Kaliningrad 236016, Russia; 3 Center for Mitochondrial Functional Genomics, Institute of Living Systems, Immanuel Kant Baltic Federal University, Kaliningrad 236040, Russia; konstantinpopadin@gmail.com; 4 Center for Integrative Genomics, University of Lausanne, 1015 Lausanne, Switzerland; 5 Swiss Institute of Bioinformatics, 1015 Lausanne, Switzerland

**Keywords:** mtDNA copies, bariatric surgery, type 2 diabetes, obesity, ddPCR

## Abstract

Mitochondrial DNA (mtDNA) encodes core subunits of oxidative phosphorylation complexes and, as a result of intricate regulatory crosstalk between nuclear and mitochondrial genomes, the total number of mtDNA copies fits the requirements of each cell type. Deviations from the physiological number of mtDNA copies are expected to be deleterious and might cause some inherited diseases and normal ageing. We studied 46 obese patients with type 2 diabetes (T2DM) one year after a laparoscopic sleeve gastrectomy (LSG) and Roux-en-Y gastric bypass (RYGB). The results were compared with normal-weight patients without T2DM (control group 1) (body mass index (BMI) = 22.5 ± 3.01 kg/m^2^) and patients with obesity without T2DM (control group 2) (BMI = 36 ± 3.45 kg/m^2^). We detected an increase of mtDNA copy number in the cells of the buffy coat obtained from peripheral blood, sampled one year after bariatric surgery. We also found that average mtDNA copy number as well as its dynamics (before and after the surgery) are gender-specific. To the best of our knowledge, this is the first evidence for the restoration of mtDNA copy number in obese patients after LSG and RYGB.

## 1. Introduction

Human mitochondria contain double-stranded circular DNA (mtDNA) that encodes 13 proteins of oxidative phosphorylation complexes and RNA elements of mtDNA gene translation machinery [1,2]. However, it is becoming more evident that mtDNA has more informational capacity than previously imagined. In addition to the universally recognized genes [3], mtDNA may encode some functional peptides [4,5], long noncoding RNAs [6,7], and even microRNAs [8]. This implies that the functional capacity of mtDNA may be much broader than previously assumed and deviations in the normal crosstalk between nuclear and mitochondrial genome may be the cause of predisposition to numerous diseases. For example, many scientific groups have recently discovered new links between mitochondrial metabolic pathways and cell functionality [9,10]. There is also increasing evidence for mtDNA copy number shifts in patients suffering from metabolic diseases. We previously discovered that that the body mass index (BMI) negatively correlates with the number of mtDNA copies in peripheral blood buffy coat cells in patients with metabolic syndrome [11]. Herein, using a droplet digital PCR approach, we performed a follow-up study where a cohort of patients with type 2 diabetes (T2DM) one year after bariatric surgery (laparoscopic sleeve gastrectomy (LSG) and Roux-en-Y gastric bypass (RYGB)) and demonstrated that their initially low mtDNA level have returned to normal, statistically non-discernible from the healthy donors. These back-and-forth dynamics suggest a functional link between mtDNA levels and obesity.

## 2. Materials and Methods

This study included 46 patients with T2DM who were obese (11 men and 35 women, 47.67 ± 8.6 years, BMI = 48.1 ± 9.4 kg/m^2^). These patients were admitted to the Regional Clinical Hospital of the Kaliningrad Region for surgical treatment. The presence of T2DM was confirmed based on a detailed clinical and instrumental examination using the World Health Organization diagnostic criteria for diabetes mellitus and intermediate hyperglycemia (1999–2013).

The patients with T2DM presenting with obesity were stratified into groups according to the type of bariatric surgery they underwent: 21 patients underwent LSG and 25 patients underwent RYGB. The results were compared with normal-weight healthy donors without T2DM (control group 1) (8 men and 10 women, 41.8 ± 4.6 years, BMI = 22.5 ± 3.01 kg/m^2^) and patients with obesity without T2DM (control group 2) (9 men and 18 women, 42.8 ± 8.3 years, BMI = 36 ± 3.45 kg/m^2^) to assess the influence of the presence/absence of type 2 diabetes. We decided to use two control groups because, one year after surgery, the patients had an increased mtDNA but the BMI had not reached a normal level (i.e., normal-weight patients).

The technical process of performing bariatric operations was standardized according to the methodical recommendations for bariatric surgeons. RYGB was performed using the Lonroth technique.

All study participants provided informed consent to participate in a research study. The study was carried out in accordance with the World Medical Association (WMA) Declaration of Helsinki (2000) and the Protocol to the Convention on Human Rights and Biomedicine (1999). The study protocol was approved by the Local Ethical Committee of the Innovation Park of the Immanuel Kant Baltic Federal University (Protocol No. 4 from 23 October 2013).

Blood samples were collected before the bariatric surgery and a year after. DNA was extracted immediately from all peripheral blood buffy coat samples by QIAmp DNA Mini Kit (QIAGEN GmbH, Hilden, Germany), according to the manufacturer’s instructions. Droplet digital PCR reaction was conducted similarly to the previously published protocol [11]. Mediators of carbohydrate metabolism (glucose, glycated hemoglobin (HbA1c)) were measured on a CA-180 automatic biochemical analyzer (Furuno Electric Co., Ltd, Nishinomiya, Japan). Quantitative determination of insulin, ghrelin, gastric inhibitory polypeptide (GIP), glucagon-like peptide-1 (GLP-1), tumor necrosis factor receptor 2 (TNFR1), and tumor necrosis factor receptor 2 (TNFR2) was evaluated by flow fluorimetry on a automated laser analyzer (Bio-Plex^®^ 200, Bio-Rad, Hercules, CA, USA) using Bio-Plex Pro Human Diabetes 10-Plex Assay commercial test system (Bio-Rad, USA) and Bio-Plex Pro™ Human Inflammation Panel 1, 37-Plex (Bio-Rad, USA).

Verification of the normality of quantitative indicator distribution was carried out using the Shapiro-Wilk test. Because the investigated samples fit a normal distribution, the hypothesis of the equality of the mean sample values was verified using Student’s t-tests. To assess the significance of differences between independent quantitative samples that did not follow a normal distribution law, the nonparametric Kruskal-Wallis test was used. For detecting statistically significant differences between groups, a pairwise analysis was performed using the nonparametric Mann-Whitney for independent groups and Wilcoxon test for dependent groups was used. To analyze associations between mtDNA levels and BMI, we used Spearman’s rank correlation.

Correlations between the studied indices were determined using the Pearson correlation analysis. Differences were considered significant at the level of *p* < 0.05. All statistical analyses have been performed in R language (version 3.3.1).

## 3. Results and Discussion

**Figure 1:**
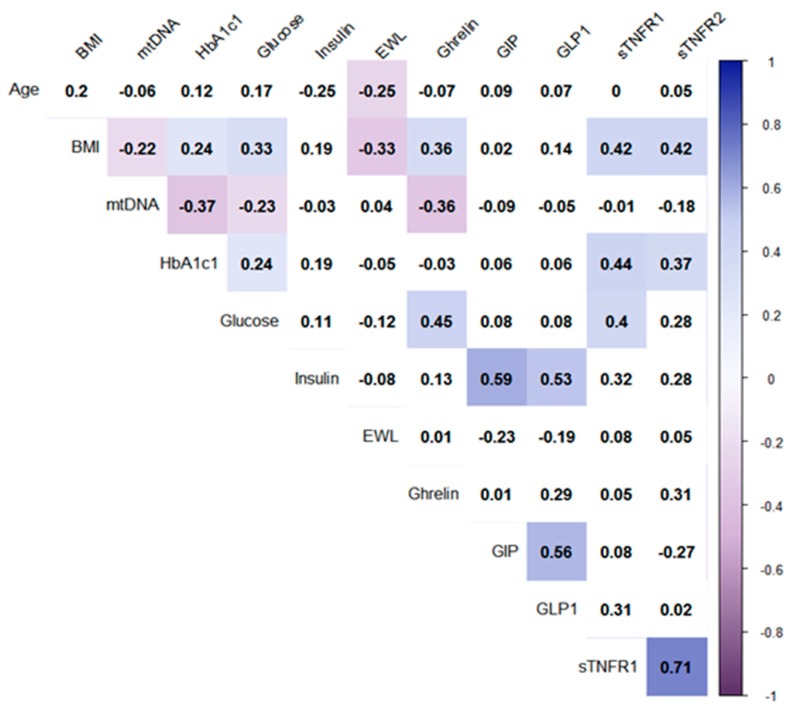
Correlations of all studied parameters (before and after surgeries) in all patients. Note: The analysis was performed using Pearson’s test correlation. Only significant correlations (*p* < 0.05) are marked by color. Intensities of the colors mark Pearson’ coefficient of correlations (from −1 to 1). Correlation matrix is quantitatively similar if we analyze whole dataset including both controls.

MtDNA copy number after surgical treatment in patients after LSG 194 (125.8–486.2) and RYGB 231 (132.0–352) after one year was higher than preoperative (*p* < 0.05, Wilcoxon test). mtDNA copies in all patients one year after bariatric operations did not differ from the ones in the control group 1. mtDNA copy number in patients before the operation of LSG was below the control (Table 1) (*p* < 0.05, Mann–Whitney U test). Thus, we observed an increase of initially low mtDNA copy number in operated patients, similar to the control groups. Moreover, in this study, mtDNA copy number was negatively correlated with the level of glucose and glycated hemoglobin (HbA1c) in blood serum of all patients (*r* = −0.24, *r* = −0.36, *p* < 0.05, Pearson’s test) (Figure 1). The link may indicate the effect of type 2 diabetes on mtDNA copy number in obese patients.

Currently there is no commonly accepted point of view concerning the link between mtDNA copies and obesity: some authors demonstrate absence of correlation between mtDNA copies and obese patients with T2DM [12]; others report a decrease of mtDNA copy number with the increase of BMI and age [13]. For example, Xu F. et al. (2012) revealed a negative correlation of mtDNA copies with age, BMI, insulin level, HOMA-IR index (Homeostasis Model Assessment of Insulin Resistance), cholesterol, and triglyceride levels in patients with T2DM [14].

Additionally, the different dynamics of biochemical parameters after bariatric surgery may determine the variability of mtDNA copy number. It is known that bariatric surgeries LSG and RYGB have fundamentally different mechanisms of influence on mitochondrial biogenesis and carbohydrate metabolism [15,16]. MtDNA copies in patients after RYGB surgery exceeded the values in control group 2 (*p* < 0.05, Mann–Whitney U test). In this regard, we investigated the relationship of mtDNA copies with the concentrations of carbohydrate metabolism components in the blood plasma of operated patients, depending on the type of bariatric surgery. All operated patients showed normalization of biochemical parameters of carbohydrate metabolism one year after surgery: the levels of glucose and HbA1c in serum decreased in patients after one year relative to preoperative values (*p* < 0.05, Wilcoxon test). The level of glucose and HbA1c in the serum of operated patients was comparable to that in the control, regardless of the type of bariatric surgery (*p* > 0.05, Mann–Whitney U test).

Insulin has the ability to modulate the synthesis of mtDNA, enhancing mitochondrial respiration and Adenosine triphosphate (ATP) production [17]. Insulin regulates the processes of glycolysis, stimulates the absorption of glucose by insulin-dependent tissues and inhibits gluconeogenesis [17]. Insulin levels decreased after RYGB and were similar to control 1, but not after LSG in patients with T2DM. In this study, mtDNA copy number was not correlated with insulin level in blood serum (*p* < 0.05, Pearson’s test) (Figure 1). The results of the correlation reject the direct participation of insulin in the regulation of mtDNA copies in patients after bariatric surgery, however, do not exclude its indirect effect on the parameters of carbohydrate metabolism.

Perhaps the increased mtDNA copies in patients after RYGB surgery (compared with a control group 2) may be interrelated with the features of postoperative hormone metabolism. The positive effects of bariatric operations LSG and RYGB on body weight and carbohydrate metabolism are achieved by changing the eating behavior and the production of certain hormones in the patients’ gastrointestinal tract. Thus, in patients after surgical treatment, the BMI decreases, however, according to literature data, restoration of insulin sensitivity precedes a significant change in the mass of adipose tissue [18]. There is an opinion that hormones such as ghrelin and incretins are primarily involved in this process.

As a result of LSG surgery, the fundus of the stomach is removed; it includes the main pool of ghrelin-producing cells [19,20,21]. Ghrelin receptor knockout animal models have shown improved sensitivity to insulin, higher thermogenesis, and mitochondrial activity by activation the AMP-activated protein kinase (AMPK) signaling pathways [22]. We found here that plasma ghrelin level was decreased in patients with T2DM after LSG compared to the pre-operating conditions (*p* < 0.05, Wilcoxon test) and correlated positively with glucose level (*r* = 0.45) and BMI (*r* = 0.36) in all observed patients with T2DM before and after bariatric surgery (*p* < 0.05, Pearson test) (Figure 1). The results may indicate the effect of ghrelin on the normalization of carbohydrate metabolism after bariatric surgery. Moreover, plasma ghrelin level correlated negatively with mtDNA copy number in all observed patients with T2DM before and after bariatric surgery (*r* = −0.36, *p* < 0.05, Pearson test) (Figure 1). The same results were observed in patients’ muscle tissue during early stages of critical illness [23]. The stimulative effect of ghrelin on mitochondria activity in neurons has been demonstrated before [24]. According to the positive correlation of plasma ghrelin level with glucose level and BMI in different patient conditions, we can assume a functional link between mtDNA copies and decreased ghrelin level after bariatric surgery. More thorough analysis is required in the future.

The secretion of GIP and GLP-1 increases after RYGB and the result of their effect on insulin may be the main reason for the decrease in glucose level [25,26]. Despite increased GIP level and no change in GLP-1 level in plasma after RYGB compared to the pre-operating condition in our study, we failed to find any correlation with patients’ mtDNA copy number. This suggests the lack of relationship between mtDNA copy number and incretins in our research.

There is evidence that obese patients with T2DM suffer from chronic subclinical inflammation of adipose tissue [27]. Moreoverr, there is the link between the BMI and the level of proinflammatory cytokines characteristic mainly for patients with T2DM [28,29]. In our previous work, we have shown the correlation between mtDNA copy number and pro-inflammatory factors (TNF-α) in such patients [30]. In line with that, we hypothesize that increased mtDNA copy number in peripheral blood buffy coat could be associated with pro-inflammatory factors circulating in patients’ blood after surgery.

Tumor necrosis factor (TNF), the superfamily TNF receptors (TNFR), and their ligands (TNFSF) include about 50 membrane and soluble proteins, all of which have a part in regulating the inflammation process [31]. The TNF family receptors have a wide spectrum of activity with the ability to regulate cell proliferation and differentiation as well as stimulate the production of inflammatory cytokines and chemokines. The binding of TNF-α to receptors (TNFR) initiates proteolytic cleavage of the latter and the formation of soluble forms called sTNFR1 and sTNFR2 [32,33]. It is assumed that the concentrations of sTNFR1 and sTNFR2 reflect the effects of TNF-α [33,34,35]. The blood plasma levels of sTNFR1 and sTNFR2 were decreased in patients after bariatric operations compared with pre-operative parameters (Table S2) (*p* < 0.05, Mann–Whitney U test), which indicates a decrease in chronic inflammation in these patients after LSG and RYGB.

The sTNFR1 plasma level was higher in patients with obesity without type 2 diabetes (control 2) compared with control 1 and compared with patients before and after LSG and RYGB (Supplementary Table S2) (*p* < 0.05, Mann–Whitney U test). An increase in the levels of sTNFR1 and sTNFR2 indicates the presence of a compensatory mechanism aimed at suppressing the effects of TNF-α in obesity [32,33,34]. We assume the presence of compensatory products sTNFR1 and sTNFR2 in patients with obesity without type 2 diabetes (control 2), and, apparently, the absence of this in patients with type 2 diabetes. Moreover, positive correlations of sTNFR1 with BMI (*r* = 0.42), glucose level (*r* = 0.4), and with HbA1c (*r* = 0.44) were found; and sTNFR2 with BMI (*r* = 0.42) and with HbA1c (*r* = 0.37) in operated patients (Figure 1) (*p* < 0.05, Pearson test). Thus, we suggest that high plasma levels of sTNFR1 and sTNFR2 may help maintain normal glucose levels in obese patients. At the same time, no correlation between mtDNA copy number and plasma levels of sTNFR1 and sTNFR2 has been found.

**Figure 2:**
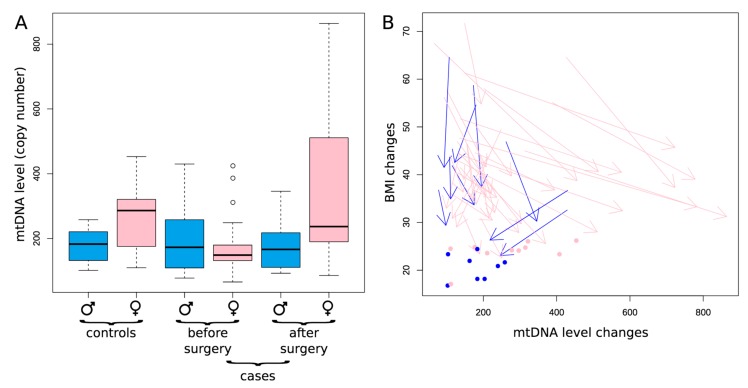
The number of copies of mtDNA in blood cells in all patients according to gender. Note: (**A**) Gender-specific variation in mtDNA level between controls and in patients before and after both bariatric operations, regardless of the type of operations. The blue boxes correspond to men, the pink ones—women; (**B**) The dynamics of changes in BMI and mtDNA level (the number of copies) is depicted for each patient by an arrow: start of each arrow corresponds to the conditions before the surgery and the end of each arrow–conditions after the surgery: blue—men, pink—women, arrows– cases, dots–controls.

Furthermore, we found out that the mtDNA copy number as well as its dynamics (before and after both bariatric surgeries: LSG and RYGB) are gender-specific (Figure 2A). For males, the level of mtDNA copies is relatively stable among the controls and cases both before and after bariatric surgery (Figure 2A, see three blue boxplots reflecting similar mtDNA level). However, for females, the dynamics is more complex (Figure 2A, see pink boxplots): cases before the bariatric surgery are characterized by the decreased mtDNA copy numbers as compared to control group 1 (*p* = 0.017), while after the surgery these values are significantly increasing (*p* < 0.001, Mann–Whitney U test) reaching the level, statistically non different from the control group 1 females (*p* = 0.64, Mann–Whitney U test) (Figure 2A). This dynamic is compatible with the hypothesis of the increase of mtDNA copy number in patients with T2DM after the bariatric operation.

To more deeply investigate the associations between mtDNA and obesity, we analyzed the dynamics of mtDNA level for each patient in the context of the corresponding changes in BMI (Figure 2B: arrows indicate the changes in the BMI and mtDNA copies before and after both bariatric operations, regardless of the type of operations). As expected, we observed decreases in the BMI in all patients: absolutely all arrows point down, which means that BMI after operation is lower than BMI before the operation. We can also see that females demonstrate an increase in the number of mtDNA copies after both bariatric operations (pink arrows, *p* < 0.01), oppositely the trend is missing for males (blue arrows, *p* = 0.55). Thus, on a patient-specific level, we can observe the increase in mtDNA copies along with a decrease in BMI, which is observable in females but not males.

Is this interconnection driven by surgery, the question arises are the BMI and mtDNA copies functionally and quantitatively connected? If there is a causative link between mtDNA level and obesity, we expect that the strength of the decrease in the BMI should correlate with the strength of the increase in mtDNA copies. From the plot (Figure 2B) we might note that pronounced changes in the BMI are associated with pronounced changes in mtDNA copies (long arrows are less steep as compared to short ones): it means that small changes in BMI are not associated with increase in mtDNA, while big changes in BMI are associated with increase in mtDNA level. To prove this observation statistically we correlated the proportional changes in the BMI (contrast in BMI for each patient was defined as BMI after the surgery minus the BMI before the surgery normalized by the BMI before the surgery) with the corresponding proportional changes in mtDNA (contrast in mtDNA for each patient was defined as the mtDNA level after the surgery minus mtDNA level before the surgery, normalized by the mtDNA level before the surgery). We observed marginally significant negative correlation between these contrasts (*p* = 0.041; rho = −0.312, one-sided Spearman rank correlation), meaning that the higher the changes in the BMI, the higher the corresponding changes in the number of mtDNA copies. This correlation of contrasts may vote towards a functional, causative relationships between the BMI and mtDNA copies.

## 4. Conclusions

Our results provide pioneering evidence for the link between changes in mtDNA copies in peripheral blood buffy coat cells and certain parameters of carbohydrate metabolism (glucose level and HbA1c) during changes in the patient’s BMI one year after bariatric surgery RYGB and LSG. The decrease in mtDNA copies may be the basic cause preceding the development of type 2 diabetes, which is consistent with our previous studies [30] and other authors’ works demonstrating the decreased mtDNA copies in patients with type 2 diabetes and the restoration of mitochondrial function and mtDNA copies after losing weight [36,37]. The mtDNA copy number is a measure of mitochondrial function and reflects the oxidant-induced cell damage [38]; it can also be used to diagnose metabolic abnormalities in patients. Another observation that will be interesting to understand in the future is why we don’t observe the mtDNA restoration effect in males. This might be due to our relatively limited sample size and initially low mtDNA level in males (even in the controls) which makes any changes less pronounced or due to a less optimal regulation of mtDNA level in males because of the relaxed selection on mtDNA properties in males [39].

## Figures and Tables

**Table 1:** The anthropometric parameters and indicators of carbohydrate metabolism in patients after laparoscopic sleeve gastrectomy (LSG) and Roux-en-Y gastric bypass (RYGB) bariatric operations. T2DM: type 2 diabetes; mtDNA: Mitochondrial DNA; BMI: body mass index.

Sample		Control Group 1 (Healthy Donors)	Control Group 2 (Patients with Obesity without T2DM)	Patients with T2DM before LSG	Patients with T2DM after LSG	Patients with T2DM before RYGB	Patients with T2DM after RYGB
Group		1	2	3	4	5	6
Anthropometric and Biochemical Parameters	Age (years)	41.8 ± 4.6	42.8 ± 8.3	48 ± 8.6	49 ± 8.6	47 ± 9.4	48 ± 9.4
	Sex (m/w)	8/10	9/18	5/16	5/16	6/19	6/19
	BMI (kg/m^2^)	22.5 ± 3.01	36 ± 3.45 *p*_1-2_ < 0.001 *	53.9 ± 8.98 *p*_1-3_ < 0.001 * p_2-3_ < 0.001 *	37.11 ± 6.22 *p*_1-4_ = 0.001 * p_3-4_ < 0.001 *	43.26 ± 7.0 *p*_1-5_ < 0.001 * p_2-5_ < 0.001 *	31.68 ± 5.2 *p*_1-6_ = 0.001 * p_5-6_ < 0.001 *
	EWL (%)	-	-	-	53.15 (40.08–62.24)	-	60.20 (47.66–70.63)
	mtDNA (copy number)	206.5 (165–228.2)	164.0 (135.7–231.5)	149.0 (115.0–180) *p*_1-3_ = 0.023 *	194.5 (125.8–486.2) *p*_3-4_ = 0.011 *	155 (132–202)	231 (185.8–352) *p*_2-6_ = 0.002 * p_5-6_ = 0.007 *
	Fasting serum glucose level (mmol/L)	4.9 (4.64–5.34)	5.89 (5.6–6.76)	8.1 (6.87–9.08) *p*_1-3_ < 0.001 * p_2-3_ < 0.001 *	4.99 (4.7–5.26) *p*_3-4_ < 0.001 * p_2-4_ < 0.013 *	8.61 (6.36–9.07) *p*_1-5_ = 0.001 * p_2-5_ = 0.013 *	5.5 (5.13–6.30) *p*_5-6_ = 0.002 *
	Insulin (pg/mL)	44.8 (32.49–54.48)	119 (55.2–193.7) *p*_1-2_ = 0.002 *	140 (105.57–454.75) *p*_1-3_ = 0.011 *	145.13 (64.5–238.22) *p*_1-4_ = 0.041 *	469.0 (161.4–767.3) *p*_1-5_ = 0.001 * p_2-5_ = 0.014 *	60.48 (36.95–303.21) *p*_5-6_ = 0.043 *
	HbA1c (%)	5.5 (3.5–5.8)	5.81 (5.6–5.9)	6.7 (6.12–7.9) *p*_1-3_ = 0.008 * p_2-3_ < 0.05 *	5.5 (4.7–6.0) *p*_3-4_ < 0.05 *	8.05 (6.5–9.9) *p*_1-5_ < 0.05 * p_2-5_ < 0.05 *	5.7 (5.0–6.6) *p*_5-6_ < 0.05 *

Note: * *p* < 0.05, differences in significance level were determined using the Wilcoxon test for two dependent samples and Mann-Whitney criterion for two independent samples; percentage of excess weight loss (%EWL); (Mean ± SD) for normal distributions (Me(Q1–Q3)) for non-normal distributions.

## Supplementary Materials

The following are available online at https://www.mdpi.com/2073-4409/8/5/430/s1, Table S1. The level of incretin and ghrelin hormones in patients after LSG and RYGB bariatric operations, [Me(Q1–Q3)]; Table S2. The level of superfamily TNF receptors in patients after LSG and RYGB bariatric operations, [Me(Q1–Q3)].
