# Supplementary material for: From Normal to Obesity and Back: The Associations between Mitochondrial DNA Copy Number, Gender, and Body Mass Index
Source: Cells. 2019 May 9;8(5):430. doi: 10.3390/cells8050430 (PMC6562937; doi:10.3390/cells8050430)
Supplement: Supplementary file 1 [file cells-08-00430-s001.pdf]

Supplementary Materials:

Table S1. The level of incretin and ghrelin hormones in patients after LSG and RYGB bariatric operations, [Me(Q1 - Q3)]

Sample	**Control group 1** (Healthy donors)	**Control group 2** (Patients with obesity without T2DM)	**Patients with T2DM before LSG**	**Patients with T2DM after LSG**	**Patients with T2DM before RYGB**	**Patients with T2DM after RYGB**
Group	1	2	3	4	5	6
Ghrelin (pg/mL)	81.14 (57.04 – 98.8)	57.1 (41.01 – 83.5)	203.38 (52.5 – 551.7)	85.01 (49.68 – 158.02) p_{3-4}=0.043 *	127.2 (101.08 – 347.2)	113.6 (62.0 – 166.5)
GIP (pg/mL)	39.6 (24.2 – 48.1)	42.3 (27.2 – 54.5)	107.64 (61.4 – 117.7) p_{1-3}<0.01 *	54.53 (37.37 – 86.85) p_{1-4}=0.017 *	115.3 (52.7 – 183.3) p_{1-5}<0.01 *	186.6 (173.5 – 229.5) p_{1-6}<0.01 * p_{5-6}=0.028*
GLP-1 (pg/mL)	13.2 (11.1 – 15.08)	5.14 (4.54 – 15.47) p_{1-2}=0.043 *	68.77 (39.27 – 79.26) p_{1-3}<0.01 *	41.46 (20.58 – 149.9) p_{1-4}=0.01 *	49.2 (27.8 – 68.8) p_{1-5}<0.01 *	58.0 (48.6 – 63.5)

Note: * - (p<0.05), differences at a significance level were determined using the Wilcoxon test for two dependent samples and Mann-Whitney criterion for two independent samples

Table S2. The level of superfamily TNF receptors in patients after LSG and RYGB bariatric operations, [Me(Q1 - Q3)].

Sample	**Control group 1** (Healthy donors)	**Control group 2** (Patients with obesity without T2DM)	**Patients with T2DM before LSG**	**Patients with T2DM after LSG**	**Patients with T2DM before RYGB**	**Patients with T2DM after RYGB**
Group	1	2	3	4	5	6
sTNFR1 (ng/mL)	112.7 (94.8 – 159.4)	257.4 (130.4 – 361.4) p_{1-2}=0.018 *	120.05 (80.6 – 138.0) p_{2-3}=0.008 *	49.2 (31.9 – 70.7) p_{1-4}<0.001 * p_{2-4}<0.001 * p_{3-4}=0.012 *	105.9 (66.05 – 141.3) p_{2-5}=0.002 * p_{3-5}=0.009 *	45.1 (30.5 – 73.2) p_{1-6}<0.001 * p_{2-6}<0.001 * p_{5-6}=0.007 *
sTNFR2 (ng/mL)	58.3 (44.1 – 73.2)	146.5 (87.1 – 179.3) p_{1-2}=0.01 *	104.6 (48.3 – 155.3)	43.02 (30.8 – 62.7) p_{2-4}=0.001 * p_{3-4}=0.038 *	72.5 (66.4 – 120.4) p_{2-5}=0.007 * p_{3-5}=0.023 *	49.8 (32.4 – 74.7) p_{2-6}<0.001 * p_{5-6}=0.026 *

Note: * - (p<0.05), differences at a significance level were determined using the Wilcoxon test for two dependent samples and Mann-Whitney criterion for two independent samples
